# Betel quid chewing as a source of manganese exposure: total daily intake of manganese in a Bangladeshi population

**DOI:** 10.1186/1471-2458-11-85

**Published:** 2011-02-07

**Authors:** Shaban W Al-Rmalli, Richard O Jenkins, Parvez I Haris

**Affiliations:** 1 Faculty of Health and Life sciences, De Montfort University, The Gateway, Leicester LE1 9BH, UK

## Abstract

**Background:**

A relationship between betel quid chewing in Bangladeshi populations and the development of skin lesions and tremor has been previously reported, for people exposed to high levels of arsenic (As) through drinking contaminated groundwater. Exposure to manganese (Mn) is also known to induce neurotoxicity and levels of Mn in Bangladeshi groundwater are also high. The present study evaluates betel quid chewing as an overlooked source of Mn exposure in a Bangladeshi population.

**Methods:**

Inductively coupled plasma mass spectrometry (ICP-MS) was used to determine (1) urinary Mn levels for 15 chewers and 22 non-chewers from the ethnic Bangladeshi community in the United Kingdom, and (2) Mn levels in betel quids, its individual components and other Bangladeshi foods.

**Results:**

Betel quid chewers displayed a significantly higher (*P *= 0.009) mean Mn concentration in urine (1.93 μg L^-1^) compared to non-chewers (0.62 μg L^-1^). High levels of Mn were detected in *Piper betel *leaves with an overall average of 135 mg kg^-1 ^(range 26 -518 mg kg^-1^). The mean concentration of Mn in betel quid was 41 mg kg^-1 ^(SD 27) and the daily intake of Mn in the Bangladeshi population was estimated to be 20.3 mg/day. Chewing six betel quids could contribute up to 18% of the maximum recommended daily intake of Mn.

**Conclusion:**

We have demonstrated that Mn in betel quids is an overlooked source of exposure to Mn in humans. Chewers display a 3.1 fold increased urinary Mn concentration compared to non-chewers. The practice of betel quid chewing contributes a high proportion of the maximum recommended daily intake of Mn, which could make chewers in Bangladesh more vulnerable to Mn neurotoxicity.

## Background

The problem of As exposure in the Bangladeshi population through drinking contaminated groundwater has been well documented. Recently Mn has been identified as another element that may pose health risks in Bangladeshi populations through drinking Mn contaminated groundwater. Mn levels higher than the World Health Organisation standard (0.400 mg L^-1^) have been detected in Bangladeshi groundwater [1-3]. Although Mn is an essential element for human health, exposure to high levels of Mn can induce neurological effects such as manganism which is characterised by movement disturbances similar to that observed in Parkinson's disease [4]. Mn can accumulate at the cellular level in mitochondria, where it disrupts oxidative phosphorylation and increases the generation of reactive oxygen species (ROS) [5]. Unlike arsenic, the source of additional Mn exposure through food consumption has not as yet been addressed for the Bangladeshi population.

Some studies have reported a relationship between Mn exposure through drinking water in Bangladesh and children's intellectual performance [6] and infant mortality [2]. More recently Ljung et al. [3] investigated Mn levels in drinking water and its relationship to various biomarkers during early pregnancy for Bangladeshi women. Although the authors investigated the relationship between Mn intake from water and Mn concentrations in urine and blood, no strong correlation was found. A possible explanation for the absence of such correlation was that the additional Mn intake from foods was not taken into consideration. For example, the habit of betel quid chewing is widely prevalent in Bangladesh [7] and the possibility that it may contribute to increased exposure to high levels of Mn has not been addressed previously.

Betel quid chewing has been practised for hundreds of years, with more than 10% of the world's population chewing betel quid daily [8]. The betel quid is commonly referred to as 'paan' in South Asian countries, including Bangladesh. The main constituents of a betel quid are *Piper betel *leaves and areca nut (the seed of the *Areca catechu *plant). It is made by wrapping chopped areca nut in a *Piper betel *leaf, and some lime (calcium hydroxide) and tobacco leaves (called *shada *in Bangladesh) or *zarda *(flavoured tobacco) may be included to improve the taste. *Zarda *is often used in betel quids instead of untreated tobacco leaves and is available in small packets or tins. A relationship between betel quid chewing and increased risk of As induced skin lesions has been reported by several workers [9-11]. Betel quid chewers who drink As contaminated groundwater have been reported to display greater skin lesions and tremor compared to non-chewers [12].

It is currently unknown if the adverse health effects observed for populations in Bangladesh who drink As and Mn contaminated water, may be exacerbated by the habit of chewing betel quids. Previously it has been suggested that arecoline, the principal organic compound in areca nut, may induce oxidative stress that explains greater incidence of skin lesions in chewers [10]. However, as yet no studies have addressed the possibility that high levels of Mn in betel quids, and possibly other dietary products, may be a contributory factor for such adverse health outcomes. To obtain information in this area, we investigated urinary Mn levels in betel quid chewers and non-chewers. We also determined the content of Mn in different foods, and especially betel quids, to compare Mn exposure in chewers and non-chewers. The daily intake of Mn from betel quids, and its contribution to the total daily intake of this element, and associated risk assessment, were determined for a Bangladeshi population. The Provisional Maximum Tolerable Daily Intake (PMTDI) and individual and combined Target Hazard Quotients (THQs) for Mn were also estimated. No previous studies have compared Mn exposure in betel quid chewers and non-chewers or reported THQ values for Mn in betel quids and other foods from Bangladesh.

## Methods

### Sample collection and study population

Ethical approval from De Montfort University, Faculty of Health & Life Sciences, Ethics committee, was obtained for a study investigating the dietary and life-style habits of different ethnic groups in the United Kingdom including members of the Bangladeshi community. Ethical approval was obtained for a questionnaire containing a series of questions regarding betel quid chewing and the volunteers were also asked to provide urine samples. The urine samples collected were kept at -20°C until further analysis. Informed consent was obtained from the volunteers prior to sample collection and questionnaire completion. The questionnaires and samples from volunteers from the Bangladeshi community were divided into two groups (betel quid chewer and non-chewers). Within the time frame of our project, it was only possible to obtain urine samples from 37 volunteers (15 chewers and 22 non-chewers although we would preferred a larger population size). Samples were collected from cities in the UK during September 2009. The age of volunteers was in the range 28 - 71 years. All these volunteers reported to be non-smokers and did not consume alcohol. The ratio of male:female was 2.5:1 for both groups combined, but was 1:1 for the chewers. Mn levels in urine were adjusted with specific gravity of urine [13]. Urine levels of Mn in non-chewer and in chewer groups were compared using a nonparametric test (Wilcoxon rank sum test) with *P *< 0.01 being considered as significant.

### Food samples

Different types of betel quid components that are widely consumed in Bangladesh (*Piper betel *leaves, areca nut, lime, tobacco and *zarda*), rice and vegetables were purchased from UK based ethnic shops in the cities of Leicester, Birmingham and London during the months of September 2008 and November 2009. Products analysed in this study mainly originated from Bangladesh, excluding some areca nut and lime, which were of Indian origin. These products are popular with Bangladeshi communities residing in the UK.

### Sample preparations

All glassware and plastic were cleaned by soaking them in 10% nitric acid (HNO_3_) for at least 12 hours and then rinsed several times with double distilled water.

### *Piper betel *leaves and other food preparation

*Piper betel *leaves were washed three times with deionised water (these leaves were also treated by washing with water in the betel quid shops), dried in an oven at 80°C for overnight, and then ground with a grinder. Other samples (areca nut, rice, tobacco and *zarda*) were also ground after drying, but without prior washing.

### Betel quid preparation

Some betel quid samples (ordinary and sweet) were collected from ethnic shops in the UK. However, betel quids were also prepared in the laboratory by combining different chewing components in proportions that are commonly used in commercial preparations. Additional information from betel quid chewers was also used for this. There is no literature data on precise quantities of the various components of betel quids that make up a typical betel quid. In our study we used one leaf (approx. 1 g dry weight), combined with areca nut (approx. 4 g), lime (approx. 0.4 g) and tobacco (0.4 g - either tobacco leaves or *zarda*). The quantities of these materials used in the quid can vary and some chewers do not include tobacco or lime in their quids. Ordinary quid contains *Piper betel *leaf, areca nut, some lime and tobacco; however, sweet quid contains similar components as the ordinary quid but with additional materials such as sweetened pan masala, coconut powder, cumin and flavoured tobacco (*zarda)*. The betel quids were dried overnight in an oven at 80°C and then ground with a grinder before digestion for analysis using ICP-MS. The dry weight of betel quids ranged from 5.5 to 11 g with an average of 7 g. This average weight, together with the mean Mn concentrations of ordinary betel quids was used for calculating the PMTDI and THQ for Mn.

### Samples digestion

#### Betel quid analysis

All chewing components including ordinary and sweet betel quids were digested by microwave assisted digestion in ultra pure 70% HNO_3 _(Romil-UpA, Ultra Purity acid). A known weight (0.2 - 0.4 g) of dried, ground, sample was mixed with 4 ml of 70% HNO_3 _and 2 ml hydrogen peroxide (H_2_O_2_) and then digested for 40 minutes using a microwave digester (Anton Paar - Multiwave 3000 Microwave Sample Preparation System, Austria) at a total pressure of 20 bars and a maximum temperature of 150°C. The power used was 800 Watts. The solution was evaporated and then made up to 25 ml in volumetric flasks with ultra pure water (Romil-UpS, Ultra Purity water) for analysis.

### Urine analysis

Aliquots (3 - 4 ml) of urine samples were digested with ultra pure 70% HNO_3 _(2 ml). The mixture was heated overnight at 90°C and then evaporated to near dryness. Subsequently, 2 ml of pure 70% HNO_3 _and 2 ml of 30% H_2_O_2 _were added to the sample and heated again for four hours. Finally the sample was evaporated and diluted with ultra pure water for analysis. Both blanks and standard reference materials were used for quality control.

### Elemental determination

Concentrations of Mn in the digested samples were determined by inductively coupled plasma mass spectrometry (ICP-MS) [A Thermo-Fisher Scientific X-SeriesII]. For instrument calibration, internal standards were used as follows: Scandium (50 μg L^-1^), Rhodium (10 μg L^-1^) and Iridium (5 μg L^-1^) in the preferred matrix of 2% HNO_3_. Also for calibration, external standards for elements were prepared in the range 0 - 100 μg L^-1 ^(ppb), both an autosampler (Cetac ASX-520) and a concentric glass venture nebuliser (Thermo-Fisher Scientific) were used. The data processing was undertaken using Plasmalab software (version 2.5.4; Thermo-Fisher Scientific, UK).

### Methodology for risk estimation

In this study two different estimated parameters were used, the Provisional Maximum Tolerable Daily Intake (PMTDI) and the Target Hazard Quotient (THQ). The PMTDI estimates the maximum daily intake of toxic element from individual food or more than one types of food; the unit used for this scale is mg of element per day. Recently, however, the United States Environmental Protection Agency (USEPA) has provided another parameter for risk assessment, the THQ, which provides a good measure of health risk assessment and estimates of non-carcinogenic effects [14]. It gives the ratio of exposure dose to the reference dose, and values of THQ greater than 1 is considered to be of concern. For our estimations, the average adult body weight of a Bangladeshi person was taken to be 68 kg [15].

Information regarding consumption of different foods by Bangladeshis was taken from the literature. It has been reported that Bangladeshis (in Bangladesh) consume on average 400 g and 650 g of raw rice per day for males and females, respectively [16]. In contrast, Bangladeshis residing in the UK consume on average 180 - 300 g of cooked rice per meal, and they generally have two meals per day [17]. Bangladeshis in UK therefore consume between 360 - 600 g cooked rice per day. Taking into consideration the moisture content of cooked rice, this is equivalent to 160 -280 g of uncooked rice. In our study, we selected a more conservative quantity of rice intake for both Bangladeshis residing in Bangladesh and in the UK. Thus we chose 500 g (for Bangladeshis in Bangladesh) and 240 g (for Bangladeshis in the UK) of uncooked rice per day and these values were used for calculating daily intake of Mn from rice consumption. The volume of water consumed by the Bangladeshi population has been reported to be 2.7 litres per day [18]. For estimating intake of Mn from tea infusions, three cups of tea per day per person was assumed, with a volume of 237 ml per cup [19]. Quantities of other foods consumed by the Bangladeshi population, residing in Bangladesh, were taken from Zablotska et al. [20]. The intakes of foods by UK Bangladeshis were calculated using data from the UK food standard agency (FSA) [21] with additional data from our own analysis of tea infusions.

### Correlation between TDI of Mn and urinary Mn levels

The total daily intake (TDI) of Mn and urinary Mn levels were taken from the literature for UK, USA, Germany, Japan, Pakistan and India (Mumbai) [22-27]. For the Bangladesh population, the TDI was calculated in-house. Urinary Mn levels for the Bangladeshi population residing in Bangladesh were taken from Ljung et al. [3]. Since several values for Mn intake are available for a particular country, for our TDI graph we selected values - if available - where both the Mn intake and urinary Mn excretion were reported in the same paper. The correlation between TDI and Mn urinary levels was calculated using SPSS software (version 17).

### Quality control and standard reference material

In this study, all the sample masses were measured to an accuracy of ± 0.1 mg. Mn concentrations determined by ICP-MS were evaluated by the use of certified reference materials and were found to be in good agreement with the certified values of the reference material. The analytical procedure and the reliability of the digestion process were validated by analysis with each measurement blank, NIES Certified Reference Material (No. 10 Rice Flour-Unpolished, Japan) and NIST standard reference material (tomato leaves NIST 1573a SRM, USA). The average recovery of Mn from the references materials were 92% and 94% of the certified values (certified values are 31 ± 1.6 and 246 ± 8 mg kg^-1^, respectively). For urine analysis, standard reference urine (Seronorm trace elements urine) was used and the recovery was 105% of certified values (certified value is 12.3 μg L^-1^).

## Results

### Urinary Mn levels in UK Bangladeshi betel quid chewers and non-chewers

**Figure 1:**
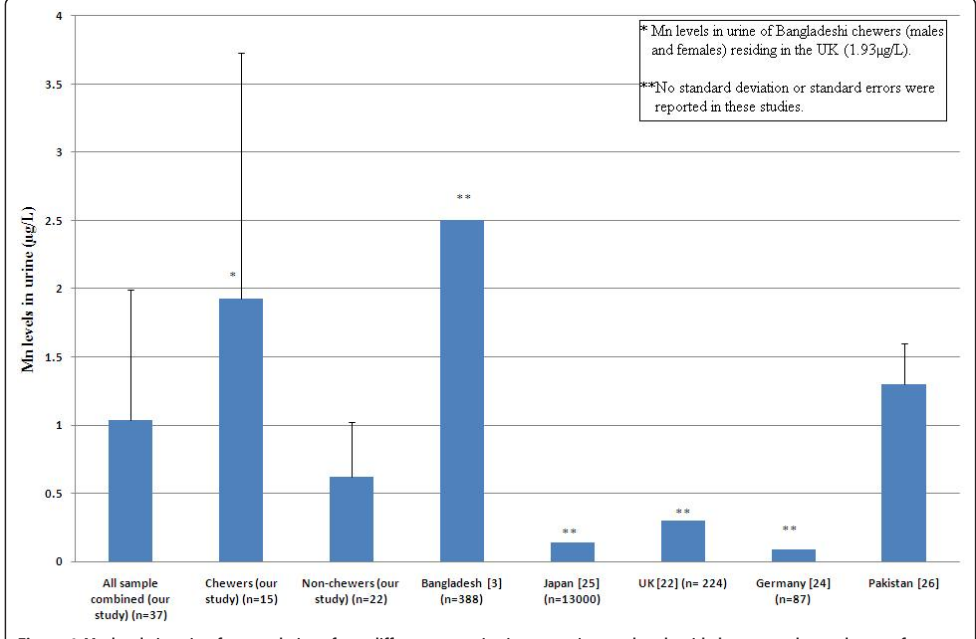
Mn levels in urine for populations from different countries in comparison to betel quid chewers and non-chewers from our study.

Mn levels in 37 urine samples from ethnic Bangladeshis living in the United Kingdom were analysed. The mean Mn concentration in all urine samples collected (chewers and non-chewers combined) was 1.04 μg/L, with a median of 0.45 μg L^-1 ^and range of 0.08 - 5.2 μg L^-1^. The mean urinary Mn levels in chewers (1.93 μg L^-1^, SD 1.8) was significantly higher (3.1 fold; *P *= 0.009 compared to non-chewers (0.62 μg L^-1^, SD 0.4) (see Figure 1). From the questionnaire, we found that an average of 3.5 betel quids was chewed by the volunteers, with a range of 1 - 30 per day.

### Mn levels in betel quids & its components

ICP-MS determination of the concentration of Mn in betel quids and its various components are shown in Table 1. The highest concentrations of Mn were found in *Piper betel *leaves (518 mg kg^-1^). However, with regards to mean concentration of Mn, the decreasing order of concentration (mg kg^-1^) was: tobacco leaves (367); *Piper betel *leaves (136); and z*arda *(flavoured tobacco; 102). The content of Mn in lime (calcium hydroxide) was considerably lower at 40 mg kg^-1^. Areca nut had the lowest concentration of Mn (12 mg kg^-1^). Ordinary betel quid contained on average 41 mg Mn kg^-1^, compared to 19 mg kg^-1 ^for sweetened ones.

**Table 1:** Concentration of Mn (mg kg^-1^) in betel quid chewing components^a^

Component	n	Mean	SD^b^	10%	25%	50% (Median)	75%	90%
Ordinary Betel quid	26	41	27	12.5	19.8	30	64.3	105.8
Sweetened Betel quid	12	19	10	10.5	13.9	16	19.5	38.7
*Piper betel *leaves	30	136	106	36.4	46.2	145	156	311.4
Areca nut	7	12	5	8.6	8.7	13	14.8	-
Lime (Chuna)	6	40	8	30.5	31.3	42	48.2	-
Tobacco	2	367	140	-	-	367	-	-
*Zarda *(flavoared tobacco)	13	102	61	19.3	46.1	97	143.5	197.8

^a ^For all samples duplicate measurements were performed.

^b ^SD: standard deviation

### PMTDI and THQ of Mn for betel quid chewing

In order to determine the PMTDI and THQs of Mn for the total diet in the Bangladeshi population, the mean consumption of different Bangladeshi foods (including meat, rice, vegetables and water) were taken partly from our own analysis and partly from the literature (Table 2). Rice samples from Bangladesh were analysed for Mn content. Levels of Mn in other important dietary intake, such as drinking water and tea infusions from Bangladesh, were taken from the literature [1,19]. These data were used for daily Mn intake estimation (Table 2). The contents of Mn in different foods, tea infusions, betel quids and water were also used for estimating PMTDI and THQ for Mn.

**Table 2:** Daily intake of Mn from foods and water (Bangladeshis residing in Bangladesh)

Food	Mn intake (μg person^-1 ^day^-1^)
*Bangladeshi foods *^*a*^	
Meat	58
Poultry	27
Fish	141 (405)^b^
Green vegetables	2751 (5400)^b^
Lentil and beans	1285
Puffed rice	168
Steamed rice	5465
Tea infusions	4168
Others	2500
*Sum of foods*	16564
Water ^c^	2160
*Total Mn intake excluding betel quid*	18724

^a ^The quantity of food consumed (g day^-1^) was taken from a previous study [20].

^b ^Mean of element concentration (maximum value).

^c ^Estimated from Frisbie et al. [1]

The Bangladeshi population (residing in Bangladesh) consumes between 400 - 650 g of uncooked rice per day [16]. In our study, the consumption of uncooked rice was assumed to be 500 grams per day. Mn levels in Bangladeshi groundwater can vary from region to region, but unfortunately information on this is not widely available. For our calculations we have used the Mn levels reported by Frisbie et al. [1]. This was combined with our data for intake of Mn from consumption of betel quids and foods to determine the total daily intake of Mn in Bangladesh. Table 3 shows the percentage of PMTDI for Mn associated with consumption of betel quids. Betel quid chewing in a Bangladeshi population has been reported to be within the range 5.7 - 6.3 quids per day [12]. Therefore, our calculations are based on an average of six quids per day.

**Table 3:** Percentage of Provisional Maximum Tolerable Daily Intake (PMTDI) for Mn associated with chewing betel quids

PMTDI (μg kgbw^-1^-day) ^a^	PMTDI (μg day^-1^)^b^	Percentage of PMTDI for Ordinary betel quid ^c^			
		3 quids	6 quids	10 quids	30 quids

140	9520	9	18	30	90

^a ^Microgram of Mn per kilogram body weight per day.

^b ^Daily intake of Mn assuming the body weight for a Bangladeshi adult is 68 kg.

^c ^Ordinary betel quid is more commonly consumed by chewers.

The percentage of Mn contribution from individual betel quid components are as follows: 30% from *Piper betel *leaves, 41.8% from tobacco leaves, 12.2% from areca nut, 11.4% from *Zarda*, and 4.4% from lime. These results show that the vast majority of Mn comes from *Piper betel *leaves and tobacco leaves.

**Figure 2:**
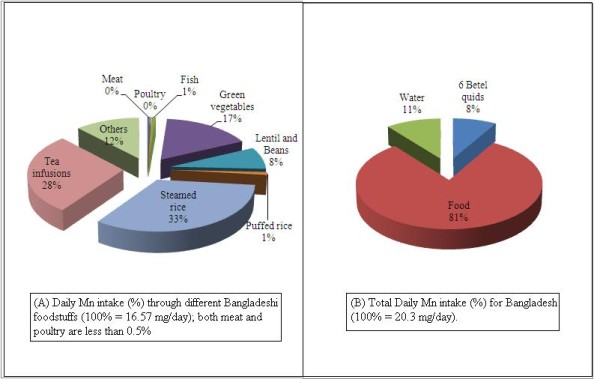
Percentage of daily Mn intake through different Bangladeshi foodstuffs.

Assuming a daily consumption of 6 betel quids and two meals of rice and other foods, the Mn intake equals 174% of PMTDI (see Figure 2). This percentage increases further if drinking water is included in the daily intake estimation. Mn intake from drinking water can contribute towards 22.7% of PMTDI which is similar to the intake from chewing six betel quids (18% of PMTDI) (Figure 2 and Table 3).

THQs for Mn in individual betel quid and for all dietary intakes in Bangladesh were estimated. The THQ for Mn from betel quid was 0.18 (for 6 quids per day), which is a significant value and is similar to that from drinking water (0.21). THQs for rice and tea infusions were 0.55 and 0.37, respectively. The THQ for Mn derived from all foods is higher at 1.55. The THQ for the total Bangladeshi diet (residing in Bangladesh) was estimated to be 1.94 including water and 6 betel quids, which is almost 2-fold higher than the maximum desirable THQ.

### Correlation between TDI of Mn and Mn urinary levels

**Figure 3:**
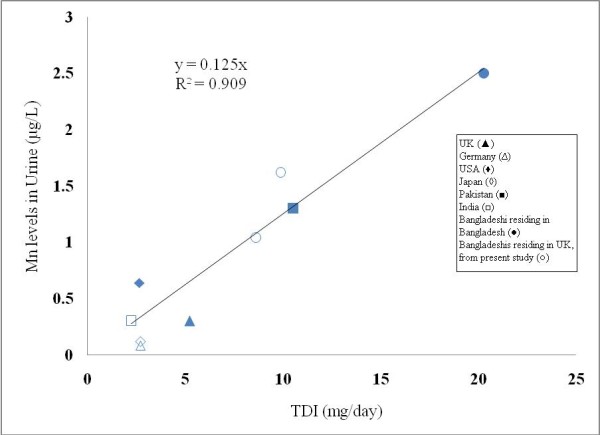
Correlation between Total Daily Intake (TDI) of Mn and urinary Mn levels for different countries.

Figure 3 shows a strong positive correlation (Pearson correlation, *P *= 0.001) between the total daily intakes (TDI) of Mn (mg day^-1^) and the urinary excretion of Mn (μg L^-1^). The TDI of Mn derived from the literature are presented in Table 4.

**Table 4:** Estimation of daily dietary intake of Mn by different populations around the world

Estimation method	Mn mg day^-1^	References
UK total diet study, healthy adult consumers (UK)	4.50	Ysart et al. (1999) [32]
UK total diet study, healthy adult consumers (UK)	5.24^a^	FSA (2009) [21]
Duplicate meals, healthy individuals (France)	2.15	Noel et al. (2003) [33]
Duplicate diet tech., healthy individuals (Austria)	4.69	Wilplinger et al. (1999) [34]
Duplicate portion tech., Healthy men (Germany)	2.70^a^	Schafer et al. (2004) [35]
DPTech, Healthy women (Germany)	2.40	Schafer et al. (2004) [35]
DPTech, Vegetarian Healthy women (Germany)	5.50	Schafer et al. (2004) [35]
Healthy adults (Catalonia, Spain)	2.23	Marti-Cid et al. (2009) [36]
Healthy adults (USA)	2.66^a^	Greger et al. (1990) [23]
Market basket study, healthy adults (Japan)	2.72^a^	Aung et al. (2006) [37]
Duplicate diet, Healthy adults (Pakistan)	10.54^a^	Iyengar et al. (2002) [43]
Healthy adults (Mumbai, India)	2.21^a^	Tripathi et al. (2000) [27]
Daily dietary intake (Murshidabad, India)	8.72	Roychowdhury et al.(2008) [44]
Healthy women (Punjab, India)	8.98	Kawatra (2008) [45]
Diet study of Bangladesh excluding water, US data based	16.51	Zablotska et al. (2008) [20]
TDI of Bangladeshi population for non chewers (Bangladesh)	18.7	Present study
TDI of Bangladeshi population for chewers (Bangladesh)	20.30^a^	Present study
TDI of Bangladeshi population for non chewers (UK)	8.64^a^	Present study
TDI of Bangladeshi population for chewers (UK)	9.88^a^	Present study

^a ^These data were used in Figure 3 for correlation between TDI and urinary Mn levels

## Discussion

Although the exposure to Mn from drinking water by Bangladeshi population has been recently highlighted [1], studies on Mn intake from foods are limited. The first study to address Mn intake from foods in the Bangladeshi diet was reported by Zablotska et al. [20], although this study did not incorporate contributions from betel quids and water. The observation by Hafeman et al. [12] that betel quid chewers, who are likely to be consuming As and Mn contaminated drinking water, had higher incidence of skin lesions and tremor led us to determine the urinary levels of Mn in chewers compared to non-chewers and also to carry out a more comprehensive estimation of the dietary intake of Mn that took into consideration contributions from betel quid chewing, tea drinking etc.

Since the Bangladeshi community in the United Kingdom have a similar diet to people living in Bangladesh [28], we determined the level of Mn in urine from 37 volunteers in this community. Betel quid chewers have a statistically significant (*P *= 0.009) higher Mn levels compared to non-chewers, which is likely to be due to the high Mn content in betel quids. Urinary Mn levels in the Bangladeshi population residing in Bangladesh have been determined by Ljung et al. [3]. They analysed urine from 388 Bangladeshi women and reported a mean concentration of 2.5 μg L^-1 ^with a median value of 1.6 μg L^-1^. Interestingly, this is similar to Mn level in urine for the male betel quid chewers in our study (2.25 μg L^-1^), but is approximately 1.5-fold higher than that for the female chewers in our study (1.62 μg L^-1^). The average urinary Mn level in the UK Bangladeshi community (chewers and non-chewers combined, 1.04 μg L^-1^) is 3.4-fold higher compared to the general UK population (0.3 μg L^-1^) [22] and is also far higher than those reported for other countries, including USA, Germany and Japan [23-25]. Although the higher urinary Mn for population residing in Bangladesh may partly arise from Mn in drinking water, this is not the case for UK Bangladeshis where the Mn in drinking water is relatively low at < 50 μg L^-1 ^[29]. Thus, the similarity between the urinary Mn levels in UK Bangladeshi chewers and Bangladeshi females (residing in Bangladesh) [3], can be mainly attributed to Mn intake from betel quids although contribution from other food sources cannot be ruled out.

The lower urinary Mn levels in UK Bangladeshi females could be due to lower betel quid consumption in the UK compared to Bangladesh and other dietary differences. Thus, for example, our questionnaire data revealed 3.5 betel quids are consumed on average per day by Bangladeshis in the UK, compared to 5.7 - 6.3 in Bangladesh [12]. Bangladeshis residing in Bangladesh have a greater proportion of vegetables in their diet compared to UK Bangladeshis who consume a higher quantity of animal products such as poultry, fish and meat. In addition, the rice intake for UK Bangladeshis is almost 50% lower compared to that of Bangladeshi's residing in Bangladesh. Thus, the higher Mn levels for the latter group can be attributed mainly to a higher intake of plant based products which are known to have greater Mn content compared to animal products. Of course Mn intake from consumption of Bangladeshi groundwater is another factor explaining the higher Mn levels for the Bangladeshi females. Ultimately, Mn levels in chewers and non-chewers residing in Bangladesh needs to be carried out to ascertain our conclusions based on studies on Bangladeshis residing in the UK.

Mn intake by pregnant Bangladeshi females is of particular concern as Mn can be easily transferred from the mother to the foetus via the placenta, and subsequently crossing the blood-brain barrier of the underdeveloped brain affecting neurodevelopment [30]. In this context, it has been reported that children exposed to Mn through drinking contaminated water in Bangladesh displayed poor intellectual function [31].

We have shown that a positive correlation exists between the total daily intake of Mn and urinary excretion of Mn for different populations (Figure 3). This is an interesting finding and such a correlation may be used for prediction of either urinary Mn excretion or total daily intake of Mn for other population, provided data on one of these two parameters are available. The positive correlation also suggests that bioaccessibility and bioavailabilty of Mn from different dietary sources, including drinking water, are likely to be similar. Our study shows that the average intake of Mn for Bangladeshi population is 18.3 and 18.7 mg day^-1 ^from foods when betel quids (6 quids) or water (2.7 litres), respectively, are included in the daily intake estimation. When both betel quid chewing and drinking water are included in the calculation, the total daily intake of Mn is 20.3 mg day^-1 ^for all foods. These results are not too different from the daily Mn intake of 16.51 mg day^-1 ^calculated by Zablotska et al. [20], despite the fact they did not include water and betel quid intake in their calculation.

From an international perspective, the total Mn intake by the Bangladeshi population is by far the highest compared to non-occupationally exposed groups in any other country for which data has been published so far (Table 4) [32-37].

The THQs of Mn estimated for the Bangladeshi population were 0.18, 1.55 and 0.21 for betel quids, food and water, respectively. Although the "food" category shows the highest THQ, the nature of the Mn species present in foods and its bioaccessibility and bioavailability may differ to that derived from betel quids and water. Surprisingly, the THQ of Mn resulting from water and betel quids are very similar, but again the bioaccessibility and bioavaliability could be very different. The issue of Mn species, Mn bioavailability and bioaccssibility needs to be investigated in the future. Nevertheless, the total THQ of Mn from Bangladeshi diet (including food, water and betel quids) should be considered as very high (1.94) as THQ values greater than one are considered to be of concern [14]. As already pointed out, the daily intake of Mn by Bangladeshis is the highest compared to other communities thus far reported and the health impact of this requires further investigation.

Several studies have attributed betel quid consumption with the development of different diseases including oral cancer, diabetes, cardiovascular disease etc [38,39]. In the UK, Asians have the highest incidence of head and neck cancer which has been attributed to smoking and betel quid chewing [40]. As pointed out earlier, chewing betel quids was associated with a higher risk of skin lesions [9] and tremor [12] in populations exposed to high levels of As in their drinking water. Unfortunately, these studies linking betel quid chewing with certain health impacts in As exposed populations did not analyse betel quids. However, a recent study analysed the As content of tobacco used in betel quids [11]. This study only focused on As content of tobacco rather than the other substances consumed as part of the chewing material such as *Piper betel *leaves, areca nut, lime etc.

The mechanism underlying Mn induced toxicity in people exposed to high levels of Mn is poorly understood. However, it has been suggested that Mn plays a role in the generation of ROS that may result in neurotoxicity [41]. More recent studies have provided evidence suggesting that oxidative stress induced by Mn exposure can trigger apoptosis of neural stem cells [42]. In light of these studies, we hypothesise that increased Mn exposure through a combination of diet, betel quid chewing and drinking water results in oxidative stress and cellular damage that may result in Mn induced neurotoxicity in certain sectors of the Bangladeshi population. Simultaneous exposure to high levels of both Mn and As may result in increased toxicity which may explain the observation of greater tremor and skin lesions in betel quid chewers. Although, several studies have reported modulating effect of areca nut in As induced skin lesions [9-11], only Pilsner et al. [10] and Lindberg et al. [11] attempted to provide an explanation for this observation. Pilsner et al. [10] suggested that ROS generated by arecoline, a key compound in areca nut, may be responsible for the higher incidence of tremor and skin lesions in betel quid chewers. Lindberg et al. [11] suggested poorer As metabolism in female chewers may responsible for their greater arsenic induced skin effects but were unable to attribute this to a particular substance in the betel quids. We suggest that the inorganic (Mn and As) and organic (arecoline) components of betel quids, are jointly responsible for the adverse health outcomes in betel quid chewers who drink contaminated groundwater. This needs to be investigated in the future.

A limitation of the present study is that the number of volunteers for the urinary Mn analysis was rather low (37 in total) and that we were only able to conduct studies with UK Bangladeshi population, rather than with participants residing in Bangladesh who drink groundwater that is often contaminated with Mn. Another limitation is that we analysed betel quid components that were sold in the UK market and we were not able to survey the wide variety of these materials that are available in Bangladesh. In addition, Mn levels in water, rice and *Piper betel *leaves, vegetables etc can vary from region to region within Bangladesh. Furthermore, the absence of information on Mn bioavailability and bioaccessibility from foods and betel quids makes it difficult to obtain an accurate estimation of exposure levels and risk assessments. Finally, we have assumed that the entire betel quid is ingested which is very common amongst the Bangladeshi community. However, this is not the case for all individuals as some spit out the juice accumulated in the mouth, whilst others spit out the fibrous portion of the betel quid after extensive chewing. Thus, the level of Mn exposure from betel quid chewing for the latter groups may prove to be lower. We plan to address these issues in future studies.

## Conclusion

Dietary intake of Mn from betel quids has not been previously considered despite the fact millions of people in Bangladesh and India consume them on a daily basis. We have demonstrated that Mn in betel quids is an overlooked source of exposure to Mn in humans. The results of this study reveal that Bangladeshis have the highest intake of Mn compared to any other population that have been studied thus far. We hypothesise that increased tremor, and possibly skin lesions, previously reported for betel quid chewers drinking arsenic contaminated water may be related to elevated exposure to Mn and other chemicals that are present in the betel quid. We also plan to carry out studies to monitor Mn levels in betel quid chewers and non-chewers from Bangladesh who consume groundwater with varying levels of Mn and As.

## Competing interests

The authors declare that they have no competing interests.

## Authors' contributions

The study was designed by SA and PH. Mn analysis was undertaken by SA. The first draft of the manuscript was undertaken by SA and comments and changes were made by PH and RJ. All authors have approved the final manuscript.

## Pre-publication history

The pre-publication history for this paper can be accessed here:

http://www.biomedcentral.com/1471-2458/11/85/prepub
